# Radiographers' Paediatric CT Radiation Dose Consideration: A Quantitative Study at Hospitals in Urban South Africa

**DOI:** 10.1002/jmrs.70116

**Published:** 2026-07-26

**Authors:** Nompumelelo Victoria Buthelezi, Lindiwe Gumede, Portia Nkhumbuleni Ramashia

**Affiliations:** ^1^ Department of Medical Imaging and Radiation Sciences Medical Imaging and Radiation Sciences University of Johannesburg Johannesburg South Africa

**Keywords:** computed tomography, paediatric patients, radiation dose, radiology, South Africa

## Abstract

**Introduction:**

Patients undergoing CT scans face cancer risks from ionising radiation, with paediatric patients being more vulnerable due to higher radiosensitivity. Radiographers' competency in paediatric CT radiation dosing is crucial for safety, protocol adherence, education and policy. This study explored radiographers' perspectives on their competency in urban South African hospitals.

**Method:**

The study employed a descriptive quantitative survey and cross‐sectional design. The study setting comprised five urban hospitals in the Gauteng province, South Africa. Data were collected using a self‐developed questionnaire. Data were analysed with the assistance of a statistician using the Statistical Package for Social Scientists (SPSS) version 29. The researchers used non‐parametric, chi‐squared and Mann–Whitney U tests to analyse the results.

**Results:**

Fifty radiographers (40% response rate) participated, with 30% male and 70% female. While 40% had good knowledge of paediatric CT radiation doses, 46% rated theirs as average. Adherence to protocols varied, often deferring decisions to radiologists. These findings highlight gaps in specialised training and documentation, stressing the need for better education and stricter paediatric imaging protocols.

**Conclusion:**

The results of this study indicate that radiographers in Gauteng who are employed in public and private hospitals have merely average knowledge of paediatric CT radiation doses, fair radiographic practice and behaviour and good perceptions of the subject. Therefore, the study recommends enhancing training on paediatric CT imaging and enforcing strict protocol adherence to improve documentation and dose monitoring for competency.

## Graphical abstract

This descriptive quantitative survey and a cross‐sectional design present an investigation on paediatric radiation dose consideration by radiographers. Paediatric patients have a higher dose risk related to CT scan exposures. This study showed that there are gaps in specialised training and a need for stricter paediatric imaging protocols.

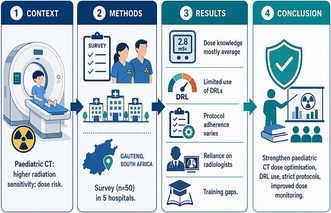

## 1 Introduction

CT imaging has become a valuable diagnostic tool, especially for paediatric patients, because of its advances [1]. The risks associated with it ultimately present significant challenges, particularly in paediatric patients [2]. Fiagbedzi et al. and Omoruyi et al. state that ionising radiation causes severe adverse reactions, such as mutations and abnormal chromosome changes [3, 4]. Some of these effects may present immediately after exposure or much later in life [3].

While scanning all patients, but especially when imaging paediatric patients, it is paramount to continuously improve CT methods and ensure reduced radiation to the patient. To reduce this radiation dose, CT protocols should thus be tailored based on the patient's age, size and clinical condition [5].

In the South African context, the responsibility of the radiographer is paramount. Mtombeni et al. emphasises that professional identity among Gauteng radiographers is closely tied to their role as gatekeepers of patient safety, yet implementation of safety protocols can be inconsistent [6]. Furthermore, recent audits in Gauteng academic hospitals by van der Merwe et al. have highlighted critical dose variations; specifically, after‐hours paediatric CT scans for infants (0–1 years) showed Dose Length Product values increasing by up to 150.56% compared to regular hours [7]. These findings underscore the urgent need to evaluate radiographer competency and adherence to protocols in urban settings.

Modern CT scanners optimise radiation doses, but awareness of potential risks in paediatric patients is lacking, despite efforts to educate radiographers [8]. Thus, this study aimed to determine and describe radiographers' perspectives on competency regarding paediatric CT radiation dose hospitals in urban South Africa.

## 2 Methods

### 2.1 Research Design

This study employed a quantitative cross‐sectional study design and used an online questionnaire to collect data. The study was conducted between 8 April 2024, and 23 May 2024.

### 2.2 Research Setting

This study was conducted at three public and two private hospitals in the Gauteng province. It is important to clarify that none of these five urban hospitals were specialised paediatric centres. This distinction is significant because the approach to paediatric imaging, including protocol rigour and specialised staffing, often differs substantially between dedicated children's facilities and general hospitals. These general facilities were selected because they provide CT services to a significant number of both adult and paediatric patients annually, making the radiographers employed there relevant to the study's objectives.

### 2.3 Population and Sampling

A census sampling method was used in this study. All radiographers who were registered with HPCSA and completed 1 year of post‐community service experience in a SA public hospital post qualification met the inclusion criteria. Those radiographers were invited to participate in the study and asked to complete the questionnaire. This census of relevant radiographers was obtained from each radiology department's assistant director/practice manager via email. The census sampling method was ideal because of the relatively small number of radiographers in the hospitals mentioned above [9].

### 2.4 Data Collection Instrument and Procedure

Data were collected using a self‐developed online questionnaire (Supporting Information) comprising four sections and 26 items. The researcher sought assistance from a Higher Education Institution (HEI) statistical consultation service, STATKON, to ensure that the questionnaire would promote the data collection process' rigour and reliability. It consisted of four sections with 26 questions collectively. Five radiographers participated in the pilot study but were not included in the main study. These radiographers met the inclusion criteria but were not employed at the institutions included in the study. No changes were made to the questionnaire after it was piloted.

The researcher requested radiographers' email addresses (after obtaining their permission to receive their emails) from the assistant director/practice manager of the organisations where the study was undertaken. The researcher blindly copied the respondents via email to request for respondents to participate in the study. A link that directed the respondents to the information letter and questionnaire was included in the same email. Clicking on the link meant that the respondents consented to being part of the study. The respondents completed the questionnaire by clicking on a link which directed them to the online questionnaire. The questionnaire took approximately 15–20 min to complete.

### 2.5 Data Analysis

The responses were anonymous, and upon completing the questionnaire, the responses were electronically loaded back onto a database that was being monitored by the statistician. The researcher used non‐parametric tests, the chi‐square and the Mann–Whitney U tests in analysing the results. The “Mann Whitney U test” was used to compare the knowledge, practice and perception of CT radiation doses among the radiographers. The chi‐square test allowed the researcher to analyse discrete data presented as frequencies since it is an independent test that helps determine the likelihood of observed relationships occurring by chance [10]. Through the descriptive results, the researcher was able to summarise respondents' demographics, their knowledge level, common practices and perceptions.

### 2.6 Ethics Statement

Ethical approval was granted by the Higher Education Institution's Research Ethics Committee (Reference No: REC‐1980‐2023). Approval was granted prior to data collection. Informed consent was obtained from all respondents.

## 3 Results

### 3.1 Demographic Data

A total of 50 respondents completed the online survey (response rate = 40%). Previous similar studies on paediatric radiation dose protocols in Gauteng had a stronger response rate of 94%. In that study, the revised questionnaires were hand delivered to each participating department by the researcher, and the researcher waited for them to be completed. In compliance with the SA Protection of Personal Information Act (POPIA) and its amended Regulations, which came into effect on 17 April 2025 [11], the researcher could not contact potential respondents directly without access to their contact details. Instead, heads of departments (HODs) and practice managers facilitated the communication by distributing the study information letter and facilitating contact with radiographers who expressed an interest in participating.

The sample comprised 15 (30%) male and 35 (70%) female radiographers. The largest responses came from the age of the 20–30 category (44%), with 38% of them having 2–5 years of experience. Only 36% of the respondents were Chief radiographers within their departments, and 74% were degree holders; a full breakdown of respondents' demographics is provided in Table 1.

**TABLE 1:** Respondents' demographics.

Variable	Groups	Respondents *n* (%)
Respondents' work experience	2–5 years	19 (38%)
	6–10 years	9 (18%)
	11–15 years	8 (16%)
	16–20 years	8 (16%)
	21 years and longer	6 (12%)
Respondents' age	20–30 years	22 (44%)
	31–40 years	14 (28%)
	41–50 years	9 (18%)
	51 years and older	5 (10%)
Gender	Male	15 (30%)
	Female	35 (70%)
Position in the radiology department	Junior Radiographers	16 (32%)
	Senior Radiographers	16 (32%)
	Chief Radiographers	18 (36%)
Respondents' education level	Diploma	12 (24%)
	Degree	37 (74%)
	Masters	1 (2%)

### 3.2 Knowledge of CT Radiation Doses and Paediatric Patients

While the study included both public and private institutions, the questionnaire did not categorise respondents by sector. Within this general hospital setting, only 46% of respondents rated their knowledge of CT radiation doses as average. When tested on specific knowledge, 64% of respondents correctly identified the primary factors to consider when imaging paediatric patients. Furthermore, 94% of respondents correctly recognised that using an adult protocol on a paediatric patient result in a high radiation dose report.

Technical knowledge regarding dose‐reduction parameters varied (Table 2). On average, 80% of the respondents correctly identified the five technical parameters used to reduce dose: kV, mA, scan time, field of view and CT protocol selection. In contrast to these high scores for technical parameters, misconceptions were noted regarding contrast media. While 65% of respondents correctly identified that contrast media is not a dose‐reduction tool, 22% incorrectly identified it as one, and 13% indicated they did not know. Table 2 summarises these findings regarding knowledge of CT radiation doses.

**TABLE 2:** Knowledge of CT radiation doses and paediatric patients.

Variable	Response category	Respondents *n* (%)
Level of knowledge of paediatric CT radiation doses	Excellent	2 (4%)
	Good	20 (40%)
	Average	23 (46%)
	Poor	5 (10%)
Paediatric CT imaging factors	Achieving high image quality	6 (12%)
	Scanning with the lowest possible radiation dose	10 (20%)
	Achieving the shortest scan times	2 (4%)
	All of the above	32 (64%)
Identification of technical parameters used to reduce CT radiation dose (Parameters rated from Used/not used/don't know)	kV	36 (72%)
		5 (10%)
		9 (18%)
	mA	34 (69%)
		6 (12%)
		10 (19%)
	Time of scan	40 (80%)
		4 (8%)
		6 (12%)
	Field of view	47 (94%)
		0 (0%)
		3 (6%)
	CT protocol	43 (86%)
		4 (8%)
		3 (6%)
	Contrast media injection	11 (22%)
		33 (65%)
		6 (13%)
Radiation dose report	High dose	47 (94%)
	Low dose	3 (6%)

### 3.3 Radiographers' Behaviour Regarding Paediatric CT Radiation Doses

**FIGURE 1:**
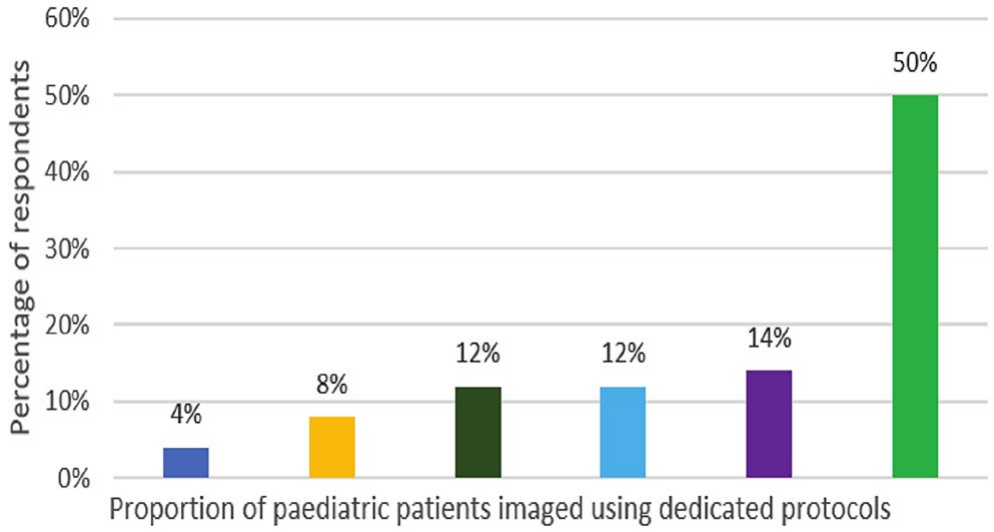
Percentage of participants according to the proportion of their paediatric patients imaged using dedicated CT protocols.

**FIGURE 2:**
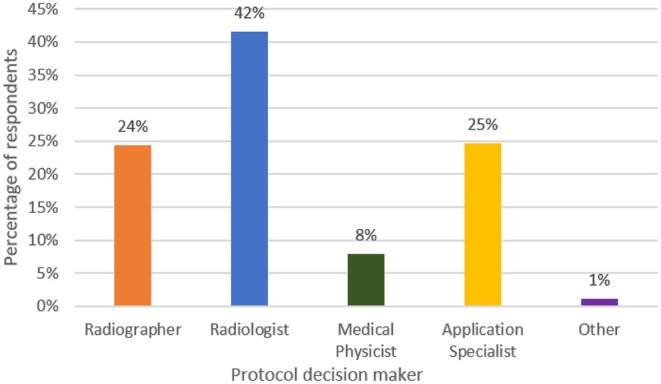
Scan protocol decision makers.

**FIGURE 3:**
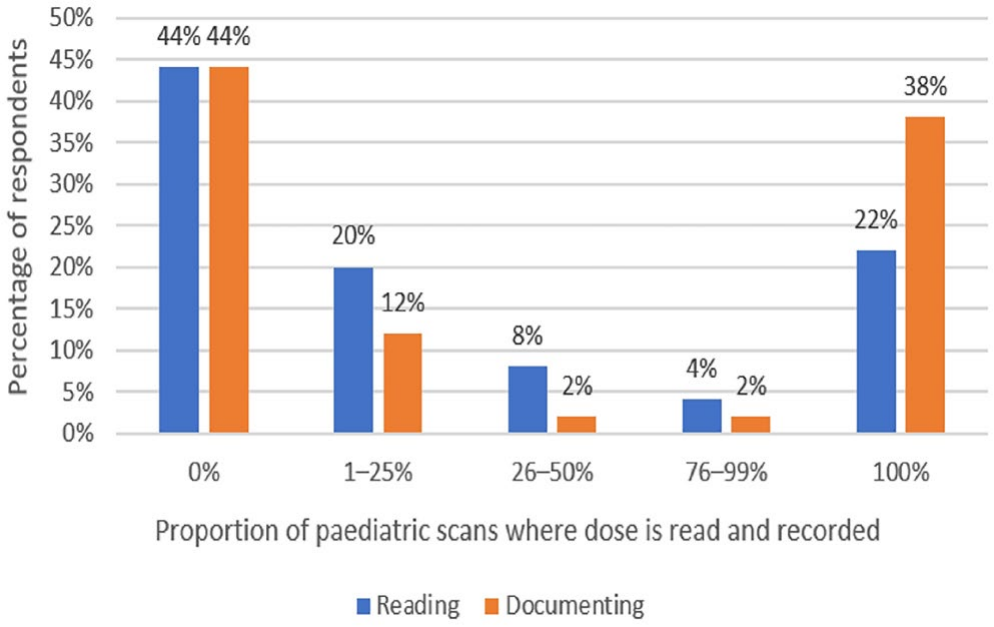
Percentage of participants grouped by the proportion of paediatric CT scans in which they read and record the dose length product.

A total of 50% of the respondents indicated they apply paediatric CT protocols to all (100%), and only a small fraction of 4% indicated that they never applied paediatric CT protocols to any (0%) patients, as demonstrated in Figure 1. More than half (55%) of respondents estimated they repeated 2% of their paediatric CT scans, while 10% estimated they repeated approximately 55% of their paediatric CT scans. A sizeable portion of the respondents (42%) indicated that radiologists are the decision makers regarding which protocol to use for patients, and only 24% stated that the radiographers make the decision, as seen in Figure 2. In addition, less than half of the respondents (44%) indicated that they did not read and document the Dose Length Product Reference for any paediatric patients received in CT, while only 38% indicated that they documented the reference; however, only 22% read the report as demonstrated in Figure 3.

### 3.4 Radiographers' Perceptions of Paediatric CT Radiation Doses and Its Effect

Only 38% of respondents strongly agreed that paediatric CT imaging is specialised imagery, while 2% indicated that they are not sure. More respondents (48%) indicated that they agree with radiographers being aware of the effects of CT radiation doses on paediatric patients and only 12% disagree. In addition, 64% of the respondents strongly agree that there is a need for specialised radiographic education on paediatric CT imaging. More than half of the respondents strongly agreed on the responsibility and importance of ensuring that appropriate CT protocols are used for paediatric patients and the importance of using paediatric‐specific protocols for paediatric patients (56% and 58%) respectively. As seen in Table 3.

**TABLE 3:** Percentage of participants' responses to statements regarding the use of paediatric‐specific CT protocols.

Perceptions of radiographers	Not Sure	Strongly disagree	Disagree	Neither agree/disagree	Agree	Strongly agree
CT paediatric imaging is specialised imaging	2%	12%	6%	12%	30%	38%
Radiographers are aware of the effects of CT radiation doses on paediatric patients	2%	2%	12%	2%	48%	34%
There is a need for specialised radiographic education on paediatric CT imaging	2%	6%	0%	2%	26%	64%
It is the radiographer's responsibility to ensure that appropriate CT protocols are utilised for paediatric patients	0%	6%	2%	4%	32%	56%
It is important to use paediatric‐specific protocols for paediatric patients	2%	8%	0%	2%	30%	58%

### 3.5 Radiographers' Practices in Paediatric CT Radiation Doses

As seen in Table 4, half (50%) of the respondents strongly disagreed that using an adult protocol on paediatric patients is acceptable. Only 36% of respondents indicated that they strongly disagreed that it is not necessary to document the DLP for all patients, 54% strongly disagreed that not considering patients' weight and age when scanning is acceptable, and 50% strongly disagreed that not shielding the gonads of the patient when scanning is considered ethical. Additionally, a total of 10% of the respondents in this study indicated that they repeat approximately 55% of paediatric CT scans.

**TABLE 4:** Percentage of participants' responses to statements regarding the use of adult protocol on paediatric patients.

CT radiation dose practices	Not sure	Strongly disagree	Disagree	Neither agree nor disagree	Agree	Strongly agree
It is acceptable to use an adult protocol on paediatric patients	2%	50%	22%	22%	2%	2%
It is not necessary to document the DLP for all patients	2%	36%	20%	18%	6%	18%
Not considering patients' weight and age when scanning is acceptable	2%	54%	32%	2%	6%	4%
Not shielding the gonads of the patient when scanning is considered ethical	2%	50%	24%	14%	8%	2%

## 4 Discussion

The findings of this study indicate the extent to which radiographers consider CT radiation dose factors when imaging paediatric patients. These factors include a thorough understanding of the risks associated with radiation exposure, dose‐reduction techniques and protection [12]. The increasing demand for paediatric CT scans highlights the need to critically evaluate existing protocols, assess potential risks and determine whether current practices require modification [13]. Competent radiographers must possess knowledge [14, 15] and practical skills [15]. Radiographers must pursue information and refine their skills to maintain professional competence [16].

### 4.1 Knowledge of CT Radiation Doses and Paediatric Patients

In this study, 64% of respondents selected the correct answer when asked to rate their knowledge of the important factors to consider when imaging paediatric patients. Most of the respondents (80%) selected the correct technical parameters used to reduce CT radiation doses (such as kV, mA and scan time), while the remaining 20% identified incorrect parameters or were unsure. However, a specific knowledge gap emerged regarding the physics of dose reduction versus image enhancement. The fact that 12.2% of radiographers were unsure and 22.4% were incorrect regarding the role of contrast media in dose reduction suggests that a third of the workforce may benefit from targeted training on the distinction between image quality enhancement and radiation dose parameters.

This finding aligns with Lewis et al., who noted that while radiographers may possess theoretical knowledge, the implementation of radiation protection varies and is heavily influenced by institutional resources and access to specialised training [17]. Furthermore, the finding from this study showing that only 38% of respondents strongly agreed that paediatric imaging is specialised suggests a potential undervaluation of the unique technical requirements for this demographic. This underscores the argument by Mtombeni et al. that professional identity and specialised education are critical components of patient safety in South African radiography, particularly in non‐specialised urban hospitals where most of these practitioners are employed [6].

### 4.2 Radiographers' Behaviour Regarding Paediatric CT Radiation Doses

The results of this study show that 50% of the respondents applied paediatric CT protocols to all (100%) of the patients they imaged. In comparison, 4% indicated they never applied paediatric CT protocols to any (0%) patients. A sum of 46% of the respondents indicated that they apply paediatric CT protocols to an average of 30% of their patients. This concurs with a study by [16], which indicated that many radiology departments continue to use adult protocols for imaging paediatric patients. A study by Garba et al. revealed that limited or insufficient knowledge of paediatric radiation dose protocols and practice has resulted in paediatric patients being considered small adults [18]. This ultimately contributes to paediatrics receiving higher radiation doses than necessary. Most of the respondents estimated repeating 2% of paediatric CT scans, which is low and shows a positive behaviour pattern. However, few studies address the repetition of scans rejected due to inadequate diagnostic quality [19]. In the present study, most respondents (41.6%) indicated that the decision for the correct or appropriate scan protocol lies with the radiologist; 24.7% indicated that the radiographer and another 24.7% indicated that the application specialist makes the decision. Only 7.9% indicated that the medical physicist made the decision, and 1.1% were unspecified. This contradicts a study by Nihan that found that it is still common practice to perform protocol decisions only by the radiographer or in collaboration with the radiologist [10]. Moolman et al. found that most CT radiographers believed protocol modification is a team approach [20]. In contrast, Bawazeer found that 59.4% of CT radiographers in their study indicated deciding themselves [21]. Almost half (44%) of the respondents in the current study indicated that they did not read and document the Dose Length Product Reference for any paediatric patients received in CT, while only 38% indicated that they document the reference; however, only 22% read the report. This aligns with the study by Bazzib et al. that reported that most radiographers did not monitor doses when scanning patients [12]. The authors indicated that radiographers are unaware of the relevance and the relationship between dose optimisation and dose survey for dose comparison.

### 4.3 Radiographers' Perceptions of Paediatric CT Radiation Doses and Their Effects

The findings regarding radiographers' perceptions of ethical practice allude to a general professional awareness of good practices within urban South African hospitals. For instance, 50% of respondents strongly disagreed that using an adult protocol on a paediatric patient is acceptable, and 54% recognised the necessity of accounting for a patient's weight and age. However, a significant discrepancy exists between these stated ethical beliefs and actual clinical performance. Despite the high level of disagreement with poor protocols, 10% of the respondents indicated that they repeat approximately 55% of their paediatric CT scans. This high repetition rate suggests that while radiographers understand the theoretical requirements for dose optimisation, practical challenges or technical errors are leading to excessive radiation exposure that contradicts the ALARA principle.

Furthermore, the finding that 50% of respondents view gonad shielding as an ethical requirement highlights a growing gap between traditional local training and evolving international standards. While radiographers in this study perceive shielding as a ‘good practice,’ recent guidelines from the American Association of Physicists in Medicine and the British Institute of Radiology now recommend discontinuing routine patient contact shielding [22]. This shift is based on evidence that such shielding can interfere with automated tube current modulation, potentially increasing the total radiation dose and degrading image quality [21, 23]. This suggests a need for updated continuous professional development to align South African practices with contemporary global evidence [24].

The respondents in the current study responded positively to the statements regarding their perceptions of paediatric CT imaging. While only 38% strongly agreed that CT paediatric imaging is considered specialised imaging, 48% of the respondents agreed that radiographers are aware of the radiation effects on paediatric patients. This perceived awareness must be contextualised by the fact that 46% of respondents in this study rated their own knowledge as only “average”. This gap between perceived awareness and consistent practice is reflected in the Gauteng‐based audit by van der Merwe et al., which found that while most diagnostic reference levels were acceptable, critical variations still exist in specific paediatric age groups [7]. Furthermore, 64% of respondents strongly agreed on the need for specialised radiographic education in CT paediatric imaging. This aligns with the observations of Lewis et al., who noted that in South African clinical settings, the successful implementation of radiation protection is heavily influenced by institutional resources and the availability of specialised training [17]. Finally, 56% of the respondents strongly agreed that it is the radiographer's responsibility to ensure appropriate CT protocols are used for paediatric patients. This strong sense of duty directly supports the work of Mtombeni et al., who found that the professional identity of radiographers in Gauteng is deeply tied to their role as responsible gatekeepers of patient radiation safety [6].

### 4.4 Radiographers' Practices in Paediatric CT Radiation Doses

A total of 50% of the respondents in this study indicated that it is unacceptable to use an adult protocol on paediatric patients. While only 18% of the respondents strongly agreed that it is necessary to document the DLP for all patients. These responses suggest a fair radiographic practice, as 50% of the radiographers offered positive feedback on their practice. The responses suggest that much needs to be done to improve the quality of CT radiographic imaging. More than half of respondents (54%) strongly disagreed that not considering patients' weight and age when scanning is acceptable, which aligns with [1], stating that CT protocols should specifically be developed for paediatric patients, considering factors such as their size, age and clinical condition. Half of the respondents (50%) strongly disagreed that not shielding the gonads of the patient when scanning is considered ethical. However, Malchair and Maccia highlighted that operators' use of gonad protection shields for paediatric patients was not consistently implemented [24].

### 4.5 Implications for Practice

The findings for this study highlight the need for continuing professional development programmes focussed on paediatric CT dose optimisation. This would help towards improving radiographer's knowledge and promote consistent application of dose reduction protocols. All healthcare institutions that provide paediatric CT services should support the implementation of standardised paediatric CT protocols including routine documentation and monitoring of radiation dose measures. Strengthening these measures could also improve patient safety and reduce unnecessary radiation exposure, thus enabling consistency in paediatric CT imaging across clinical settings.

### 4.6 Limitations

The primary limitations of this study were related to the research methodology and data collection process. The researcher did not include a section in the questionnaire to identify which sector or institution the radiographers worked in (public or private). This would have given the researcher a comparable indication of the work cultures and the radiographers' practices in both government and private institutions. Although this study did not differentiate between public and private sector respondents, the findings must be viewed through the lens of known systemic disparities. Lewis et al. found that while South African radiographers generally possess optimal knowledge of radiation protection, actual implementation is heavily influenced by institutional resources and support [17]. The resource disparities between the public and private sectors in South Africa may explain the “average” knowledge levels (46%) and varied protocol adherence observed in this study, as institutional culture often dictates the rigour of dose monitoring.

Only five out of six institutions identified for data collection permitted the researcher to collect data from its radiographers. This resulted in the anticipated sample size decreasing as many radiographers were excluded, so the results generalisation was slightly obscured.

The data collection process entailed that the researcher could not email the respondents directly as the POPI Act restricted access to their email addresses. The sample size could have likely been more significant had the researcher personally explained the study and its intention to the respondents and received a higher number of email addresses to which to distribute the link. Consequently, potential non‐response bias must be acknowledged, as radiographers with a greater interest in radiation safety may have been more likely to participate [20]. The resulting 40% response rate is notably lower than the 94% achieved by Moolman et al. in a similar study on paediatric protocols in Gauteng. This discrepancy likely stems from the recruitment method; whereas Moolman et al. utilised hand‐delivered surveys, this study relied on digital distribution due to the constraints of the POPI Act.

### 4.7 Future Research

The study's findings indicated the need for increased knowledge among radiographers regarding paediatric CT radiation doses. As a follow‐up study, the researcher could identify in which sector or institution the radiographers worked (public or private). The results of this study would allow the researcher to compare and determine the differences in the radiographers' knowledge and practice in private and state institutions. A qualitative study conducted in both sectors could provide more insight into how radiographers define competency concerning their training and exposure to paediatric CT imaging.

## 5 Conclusion

The results of this study indicated that radiographers in Gauteng, employed in both public and private hospitals, possess an average level of knowledge regarding paediatric CT radiation doses. While they demonstrate average radiographic practices and positive perceptions of paediatric CT imaging, inconsistencies remain in protocol application. Despite recognising key factors in paediatric imaging, few radiographers perceive paediatric CT as a specialised discipline, and many doubt their colleagues' awareness of the effects of CT radiation on paediatric patients. These findings underscore the necessity of enhanced education and training to improve radiographers' competency in paediatric CT imaging. The study highlights gaps in specialised training and documentation practices, reinforcing the need for standardised paediatric CT protocols and their consistent implementation.

## Ethics Statement

Ethics approval was granted by the University of Johannesburg Research Ethics Committee (Reference No: REC‐1980‐2023).

## Conflicts of Interest

The authors declare no conflicts of interest.

## Supporting information


**Data S1:** Supporting Information.

## Data Availability

The data that support the findings of this study are available on request from the corresponding author. The data are not publicly available due to privacy or ethical restrictions.
