# Supplementary material for: Radiographers' Paediatric CT Radiation Dose Consideration: A Quantitative Study at Hospitals in Urban South Africa
Source: J Med Radiat Sci. 2026 Jul 26:10.1002/jmrs.70116. Online ahead of print. doi: 10.1002/jmrs.70116 (PMC13402845; doi:10.1002/jmrs.70116)
Supplement: Supplementary file 1 — Data S1: Supporting Information. [file JMRS-9999-0-s001.pdf]

A survey of radiographer's knowledge, practices, and perceptions of paediatric computer tomography radiation doses.

We first need to establish if you meet the criteria to complete the questionnaire.

Are you registered with HPCSA?

No	
Yes	

Have you been working for more than 1 year as a qualified radiographer?

No	
Yes	

SECTION A -DEMOGRAPHICS AND BACKGROUND

1. How many years have you worked as a qualified radiographer?

☐ 2-5 years ☐ 6-10 years ☐ 11-15 years ☐ 16-20 years ☐ 21 years or longer

2. What is your age?

☐ 20-30 years ☐ 31-40 years ☐ 41-50 years ☐ 51 years or older

3. What is your gender?

☐ Male ☐ Female ☐ Other

4. What position do you hold within the radiology department?

☐ Junior radiographer ☐ Senior radiographer ☐ Chief radiographer

5. What is your highest qualification in radiography?

☐ National Diploma ☐ Degree ☐ Masters ☐ Doctorate

6. On average, how many patients do you personally image each week?

None	
1-3	
4-6	
7-9	
10-12	
13-15	
16-20	
More than 20	

7. What proportion of your paediatric patients are from each of the following age categories?

	None	1-25%	26-50%	51-75%	76-100%
2 years or younger					
2-4 years					
5-8 years					
9-11 years					
12-14 years					
15-18 years					

8. Do you have standardised CT paediatric protocols in your department/hospital?

No	
Yes	
Don't know	

SECTION B - Knowledge of CT radiation doses and paediatric patients

1. How would you rate your knowledge of paediatric CT radiation doses?

Excellent	
Good	
Average	
Poor	
Very poor	

2. What is the most important factor when imaging paediatric patients using CT SCAN?

Achieving high image quality	
Scanning with the lowest possible radiation dose	
Producing requested scans as soon as possible	
Achieving shortest scan times	

3. Which of the following parameters can be used to reduce CT radiation dose? (Mark all applicable).

**Parameter**	**Yes**	**No**
Kv		
Ma		
Time of scan		
Field of view		
CT protocol		
Contrast media injection		
4. A radiographer uses an adult protocol for a paediatric patient. Which of the following is the likely result for the radiation dose report? High radiation dose report		
Normal radiation dose report		
Low radiation dose report		

4. A radiographer uses an adult protocol for a paediatric patient. Which of the following is the likely result for the radiation dose report?

	Yes	No
High radiation dose report		
Normal radiation dose report		
Low radiation dose report		

5. When scanning a patient in CT which of these factors contribute to choosing the correct protocol to reduce radiation dose (mark all applicable)

	Yes	No
Weight of patient		
Height of patient		
Gender of the patient		
Age of patient		
Patient’s pathology		

SECTION C - Behaviour of radiographers

1. How often do you apply your paediatric computer tomography protocols?

Never	
Rarely	
Sometimes	
Often	
Always	

2. Are you capable of altering the parameters of paediatric computer tomography protocols in your department [or hospital]?

No	
Yes, some parameters	
Yes, all parameters	

3. For what percentage of paediatric patients do you have to repeat CT examinations?

None	
1-10%	
11-20%	
21-30%	
31-40%	
41-50%	
51-60%	
61-70%	
71-80%	
81-90%	
91-100%	

4. Who makes scan protocol decisions in your institution or workplace? (Mark all applicable)

Radiographer	
Radiologist	
Medical physicist	
Application specialist	
Other (please specify)	

5. How often do you read or document the Dose Length Product Reference that each paediatric patient receives in CT?

Never	
Rarely	
Sometimes	
Often	
Always	

SECTION D: Perception of paediatric computer tomography radiation doses

1. CT paediatric imaging is specialized imaging:

Strongly disagree	
Disagree	
Neither agree nor disagree	
Agree	
Strongly agree	
Unsure	

2. Radiographers are aware of the effects of CT radiation doses on paediatric patients:

Strongly disagree	
Disagree	
Neither agree nor disagree	
Agree	
Strongly agree	
Unsure	

3. There is a need for specialized radiographic education in CT paediatric imaging

Strongly disagree	
Disagree	
Neither agree nor disagree	
Agree	
Strongly agree	
Unsure	

4. If you answered question 3 as “**strongly agree**” or “**agree**” please specify the required level of education.

Continuing Professional Development (CPD)	
Postgraduate course at the university	
Other (please specify)	

5. It is the radiographer’s responsibility to ensure that appropriate CT protocols are utilised for paediatric patients

Strongly disagree	
Disagree	
Neither agree nor disagree	
Agree	
Strongly agree	
Unsure	

6. It is important to use paediatric-specific protocols for paediatric patients

Strongly disagree	
Disagree	
Neither agree nor disagree	
Agree	
Strongly agree	
Unsure	

7. South African hospitals should follow international protocols when deciding on radiation doses for paediatric patients

Strongly disagree	
Disagree	
Neither agree nor disagree	
Agree	
Strongly agree	
Unsure	

Thank you for completing this questionnaire
